# Giant Morel-Lavallée lesion complicated with open pelvic fracture: a rare case report

**DOI:** 10.3389/fsurg.2025.1639073

**Published:** 2025-08-12

**Authors:** Xiaomin Li, Rui Li, Xiangli Luo, Teng Chen, Jian Wu

**Affiliations:** ^1^ Department of Orthopedics, Wangjing Hospital, China Academy of Chinese Medical Sciences, Beijing, China; ^2^ Department of Orthopedics, Gansu Provincial People’s Hospital, Lanzhou, China; ^3^ Department of Endocrinology, Affiliated Hospital of Gansu University of Chinese Medicine, Lanzhou, China

**Keywords:** Morel-Lavallée lesion, open pelvic fracture, misdiagnosis, case report, rare

## Abstract

**Background:**

Morel-Lavallée lesion (MLL) is a rare closed degloving injury from high-energy shear/crush trauma, often linked to pelvic/acetabular fractures. Due to limited clinical awareness, it is highly prone to misdiagnosis, potentially causing severe complications like infection and skin necrosis. Existing literature rarely documents giant MLL with open pelvic fractures. This article reports such a case in an obese patient after heavy-object impact, aiming to enhance understanding and improve management of complex injuries.

**Case presentation:**

A 27-year-old male sustained lumbosacral giant MLL and open pelvic fracture from workplace heavy-object crush. The MLL was unrecognized for two weeks at another hospital. Upon transfer, he had hemorrhagic shock, fluctuating lumbosacral swelling, and localized skin necrosis. Imaging showed comminuted left iliac fracture with extensive soft tissue edema and gas.

**Management:**

A three-stage surgery was used. Stage 1: muscle debridement, fracture fixation, and vacuum-assisted closure (VAC) to address acute injury and control infection. Stage 2: debridement, antibiotic-loaded bone cement implantation, and continued VAC for osteomyelitis treatment and bone healing. Stage 3: flap reconstruction and skin grafting to restore soft tissue integrity.

**Outcome:**

The patient was discharged one month postoperatively with viable skin grafts. At 3-month follow-up, fracture and wound healed well, confirming the staged approach's effectiveness.

**Conclusion:**

MLL is often overlooked in high-energy trauma, emphasizing early comprehensive exams plus imaging to avoid misdiagnosis. For MLL with fractures, staged treatment is advisable: early radical debridement and VAC to control infection, followed by fixation and soft tissue reconstruction when stable. VAC reduces infection risk by minimizing exudation and promoting granulation. Obese patients, with loose adipose compartments, face higher MLL risk and need special attention. Future research should explore minimally invasive techniques to optimize outcomes.

## 1 Introduction

The Morel-Lavallée lesion (MLL) is a rare closed degloving soft tissue injury caused by high-energy trauma, first described in 1863 by the French physician Morel-Lavallée (1). It typically results from blunt shear or crush forces that abruptly separate the subcutaneous tissue from the underlying fascia, disrupting traversing blood and lymphatic vessels and leading to the accumulation of blood, lymph, and tissue fluid within the resulting cavity (2, 3). MLL most commonly occurs in the hip and thigh regions due to their high mobility, smooth superficial fascial surface, and sparse fibrous connections between the deep fascia and subcutaneous tissue, which facilitate separation under shear stress. Additionally, the rich capillary networks in these areas further predispose them to MLL formation (4). Clinically, MLL is uncommon and exhibits highly variable presentations, ranging from overt ecchymosis, edema, and abrasions to cases with no external signs. It frequently occurs in polytrauma patients, often alongside pelvic or acetabular fractures. Due to insufficient clinical awareness, emergency evaluations may lead to delayed diagnosis or misdiagnosis, significantly increasing the risk of complications such as infection, soft tissue necrosis, hemorrhagic shock, and pseudocyst formation (5). This report presents a rare case of a male patient who sustained an open pelvic fracture with an extensive MLL following a heavy-object crush injury, managed through a systematic surgical approach. The case adheres to the SCARE and PROCESS guidelines (6, 7).

## 2 Case presentation

**Figure 1:**
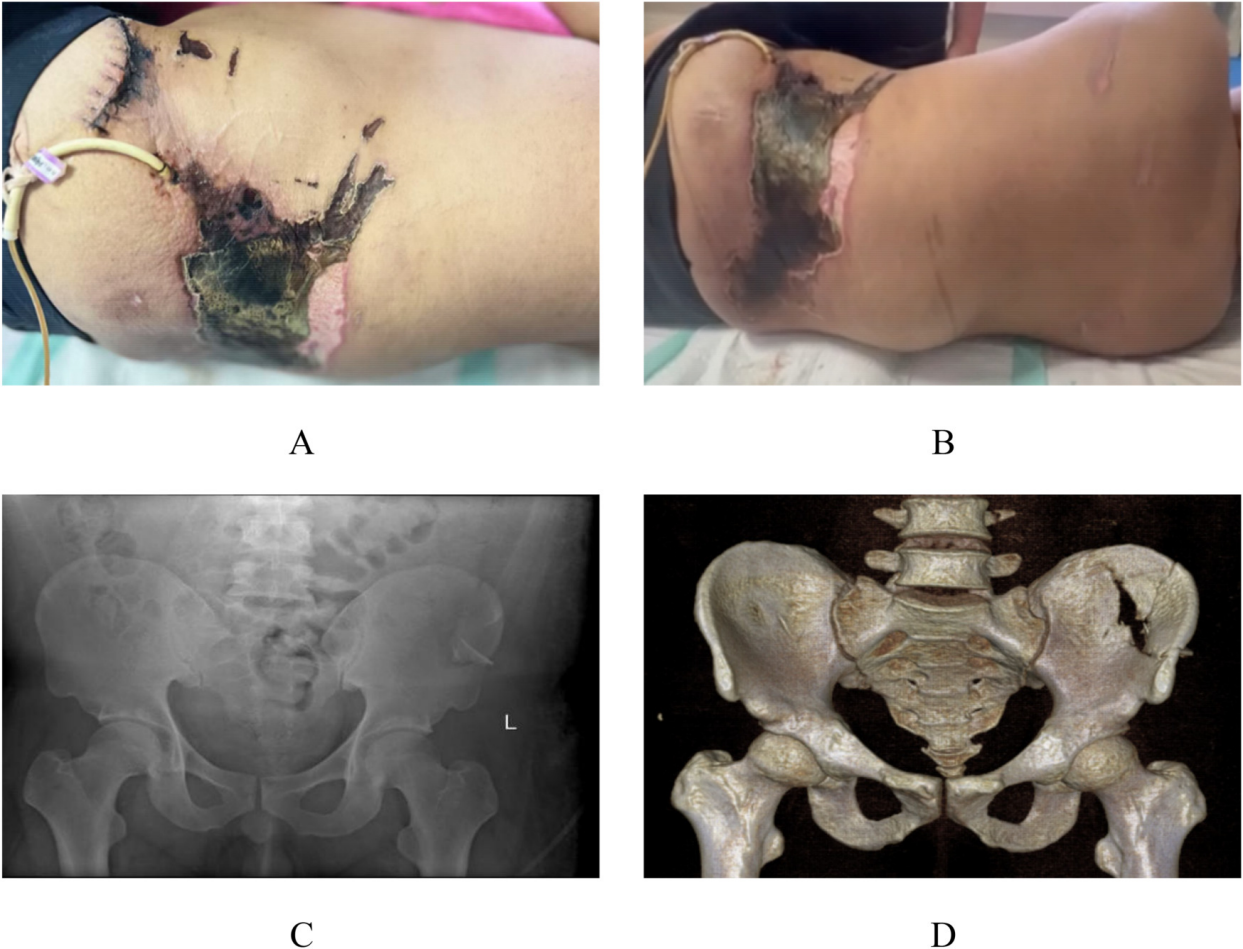
Preoperative appearance and imaging. Local clinical photographs upon admission to our hospital **(A,B)**. x-ray images from our hospital **(C)** CT images from our hospital **(D)**

A 27-year-old male was transferred to our institution for further evaluation and management of lumbosacral soft tissue injuries complicated by an open pelvic fracture and hemorrhagic shock, sustained after being struck by a swinging heavy object at a construction site. Initial CT evaluation at a local hospital revealed bilateral pulmonary inflammatory changes with dependent atelectasis, hemothorax and pleural effusion, multiple bilateral rib fractures, left adrenal gland contusion with hematoma formation, soft tissue injuries involving bilateral chest/abdominal walls and lumbosacral region, and left L3–4 transverse process fractures. Due to limited treatment capabilities at the referring center, the patient—with no significant medical history—was transferred to our facility where admission vitals showed: temperature 36.7°C, heart rate 87 bpm (regular), respiratory rate 20/min, and blood pressure 147/92 mmHg. Physical examination demonstrated an alert patient with persistent lumbosacral pain, palpable fluctuance in the lumbosacral region, a 12 cm sutured laceration over the left iliac area, and an 8 × 17 cm zone of cutaneous necrosis in the left lumbar region (Figures 1A,B), with positive pelvic compression test but otherwise unremarkable head, thoracoabdominal and extremity findings. Preoperative imaging included radiographs confirming left iliac fracture (Figure 1C) and CT scans revealing ventral abdominal wall edema with minimal fluid collection, left L2–4 transverse process fractures, comminuted left iliac fracture, bilateral gluteal edema with minimal fluid accumulation, and soft tissue gas in the left gluteal region (Figure 1D). Provisional diagnoses comprised: (1) Morel-Lavallée lesion with open pelvic fracture; (2) necrotizing fasciitis; (3) soft tissue infection; (4) type 2 diabetes mellitus; (5) anemia; and (6) electrolyte imbalance.

**Figure 2:**
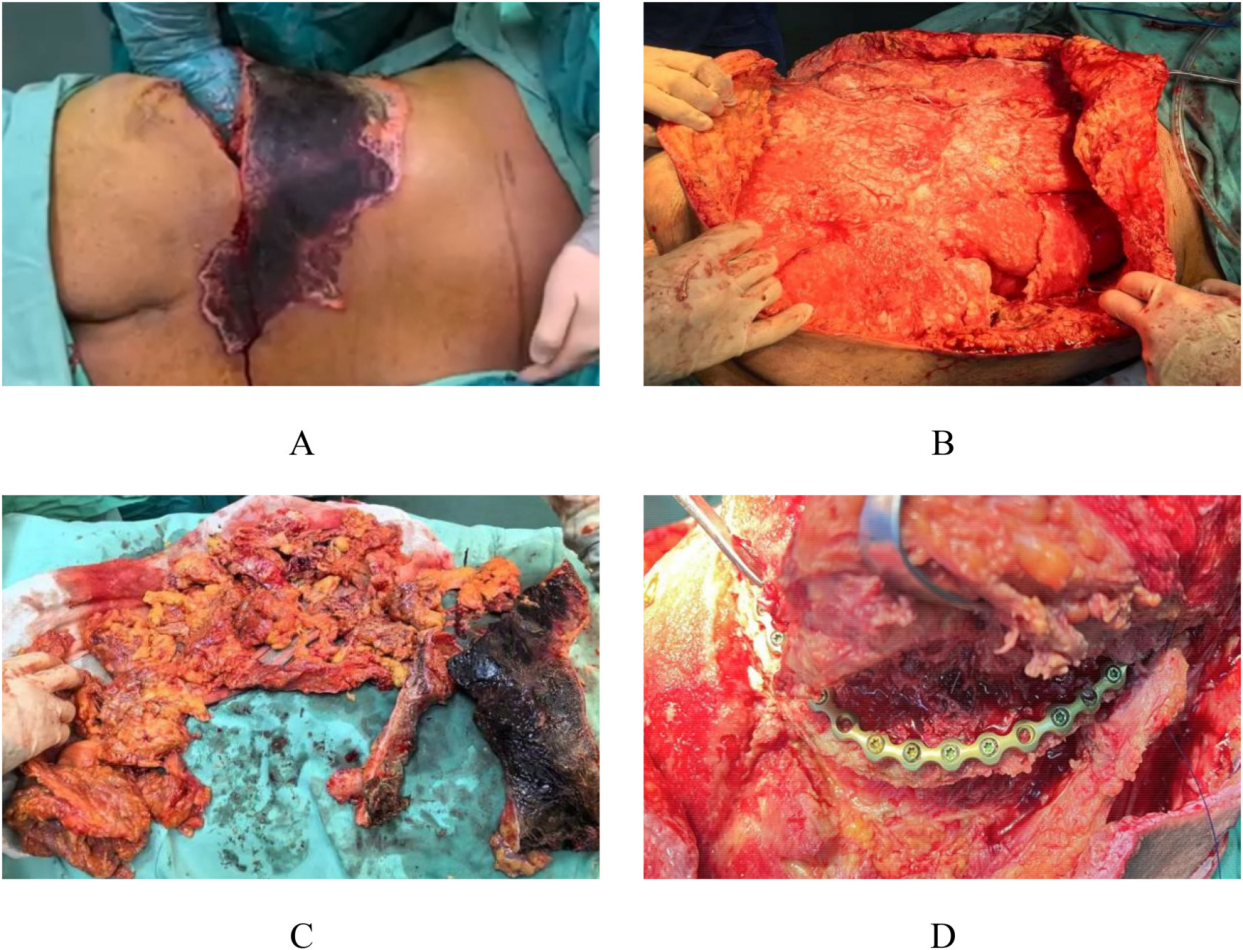
Intraoperative appearance during the first stage. Intraoperative exploration revealed extensive Morel-Lavallée lesions in the lumbar and gluteal regions **(A)** Early debridement of the injured area was performed **(B)**, with removal of infected and necrotic tissue **(C)**, followed by preliminary fixation of the iliac fracture **(D).**

Based on the patient's preoperative condition, a staged surgical management plan was implemented. The first-stage procedure included muscle debridement, iliac fracture reduction and fixation, and VAC drainage. Intraoperative exploration revealed extensive Morel-Lavallée lesions in the lumbosacral and gluteal regions (Figure 2A). Initial radical debridement was performed (Figure 2B), with complete removal of infected and necrotic tissues (Figure 2C). After performing limited internal fixation of the iliac fracture, VAC was continuously applied to reduce the risk of infection (Figure 2D), complemented by regular bedside wound care. Local bacterial culture and drug sensitivity test were performed. The results showed the growth of *Enterobacter cloacae* complex, which was sensitive to cefoperazone-sulbactam and levofloxacin, and resistant to ampicillin.

**Figure 3:**
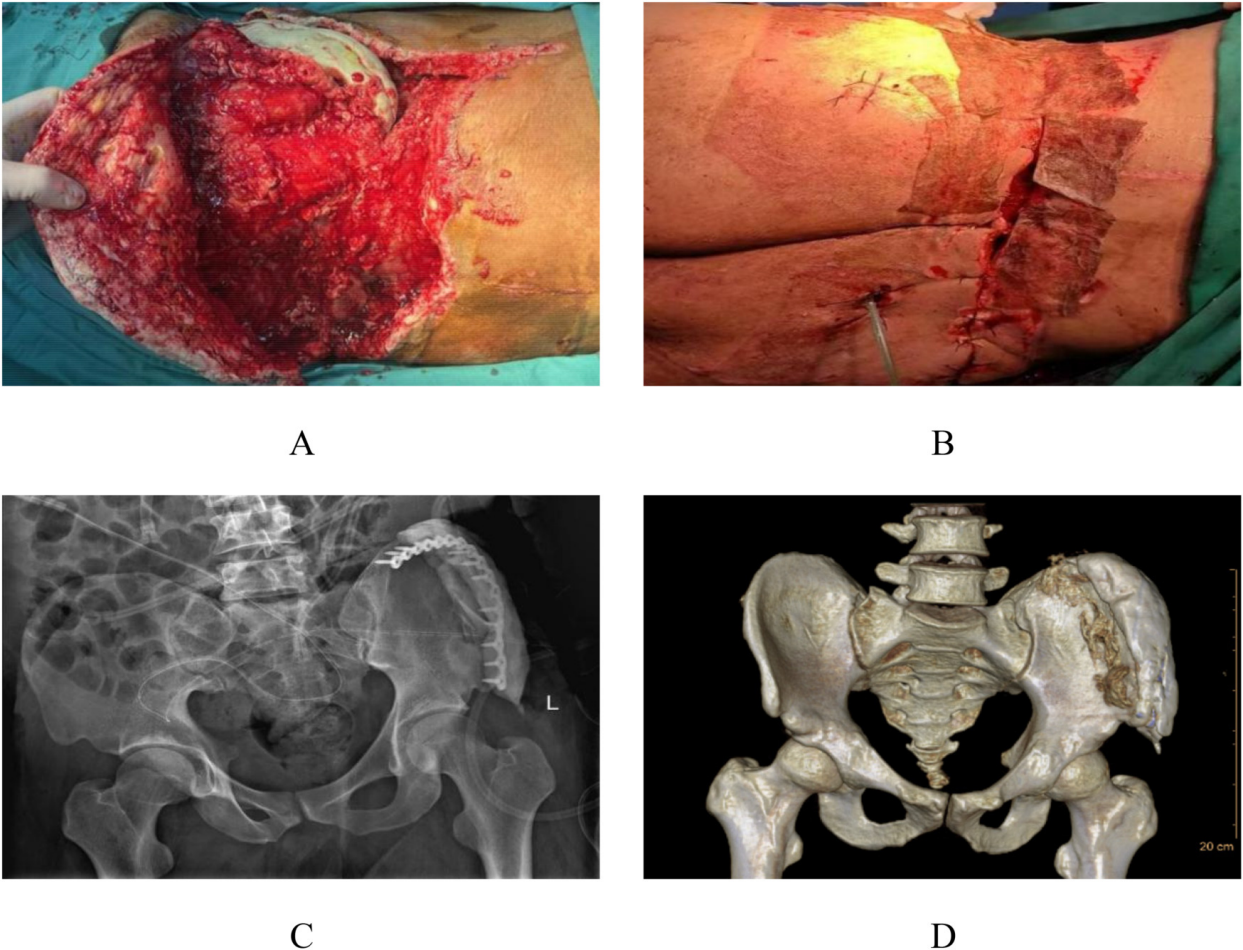
Intraoperative appearance during the second stage and postoperative imaging. The infected bone tissue was treated with antibiotic-loaded bone cement to address the bone infection **(A)**, and continuous negative pressure drainage was continued to reduce infection **(B)** A follow-up x-ray was performed 1 week postoperatively **(C)** Follow-up CT scans were conducted 1 week after the operation **(D)**.

After the acute infection subsided, the patient underwent the second-stage surgery. The second-stage surgical intervention comprised pelvic reconstruction with polymethylmethacrylate void filling and VAC drainage, initiated due to developing osteomyelitis following the initial procedure. This phase involved radical debridement of infected bone tissue followed by antibiotic-impregnated bone cement (vancomycin + tobramycin were used in this case) application for infection control (Figure 3A), with continued VAC therapy to mitigate reinfection risk (Figure 3B). One-week postoperative radiographs demonstrated maintained internal fixation of the left iliac fracture with drainage catheters *in situ* and persistent periprosthetic soft tissue swelling (Figure 3C). Follow-up CT at 7 days revealed: (1) satisfactory positioning of the left iliac comminuted fracture hardware, (2) improved bilateral gluteal soft tissue edema with minimal residual fluid collections, and (3) increased gas accumulation within bilateral gluteal soft tissues compared to prior imaging (Figures 3D), necessitating ongoing bedside wound management.

**Figure 4:**
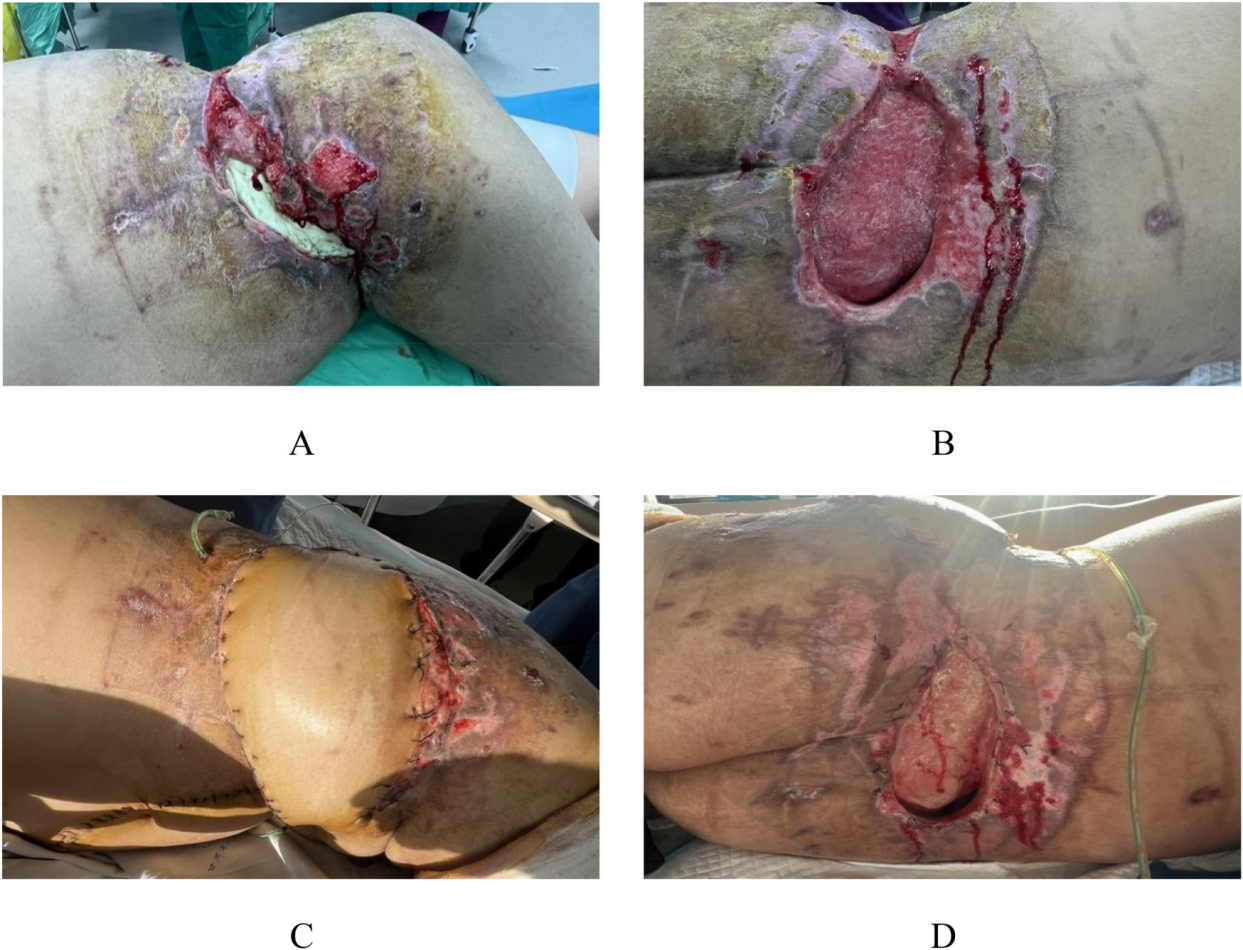
Preoperative and postoperative appearance during the third stage. In the third stage, following radical debridement, the bone cement in the left iliac spine area of the patient was exposed, and the local skin failed to heal **(A,B)**. A combined skin flap repair and skin grafting procedure was performed, transplanting the left abdominal skin flap to the lumbodorsal region **(C,D)**.

After completing soft tissue debridement and control of bone tissue infection, the third-stage surgery was initiated. The third-stage surgical management involved complex flap reconstruction (Pedicled rectus abdominis myocutaneous flap, including musculocutaneous and ultrathin flaps) combined with trunk skin grafting, additional debridement, and VAC drainage. Following thorough debridement, examination revealed exposed antibiotic-impregnated bone cement at the left iliac crest with associated wound dehiscence (Figures 4A,B). The reconstructive procedure comprised transfer of a left abdominal flap to the lumbosacral defect, supplemented by split-thickness skin grafting, followed by continued VAC therapy to optimize graft adherence and minimize infection risk (Figures 4C,D). Continuous bedside wound dressing change.

**Figure 5:**
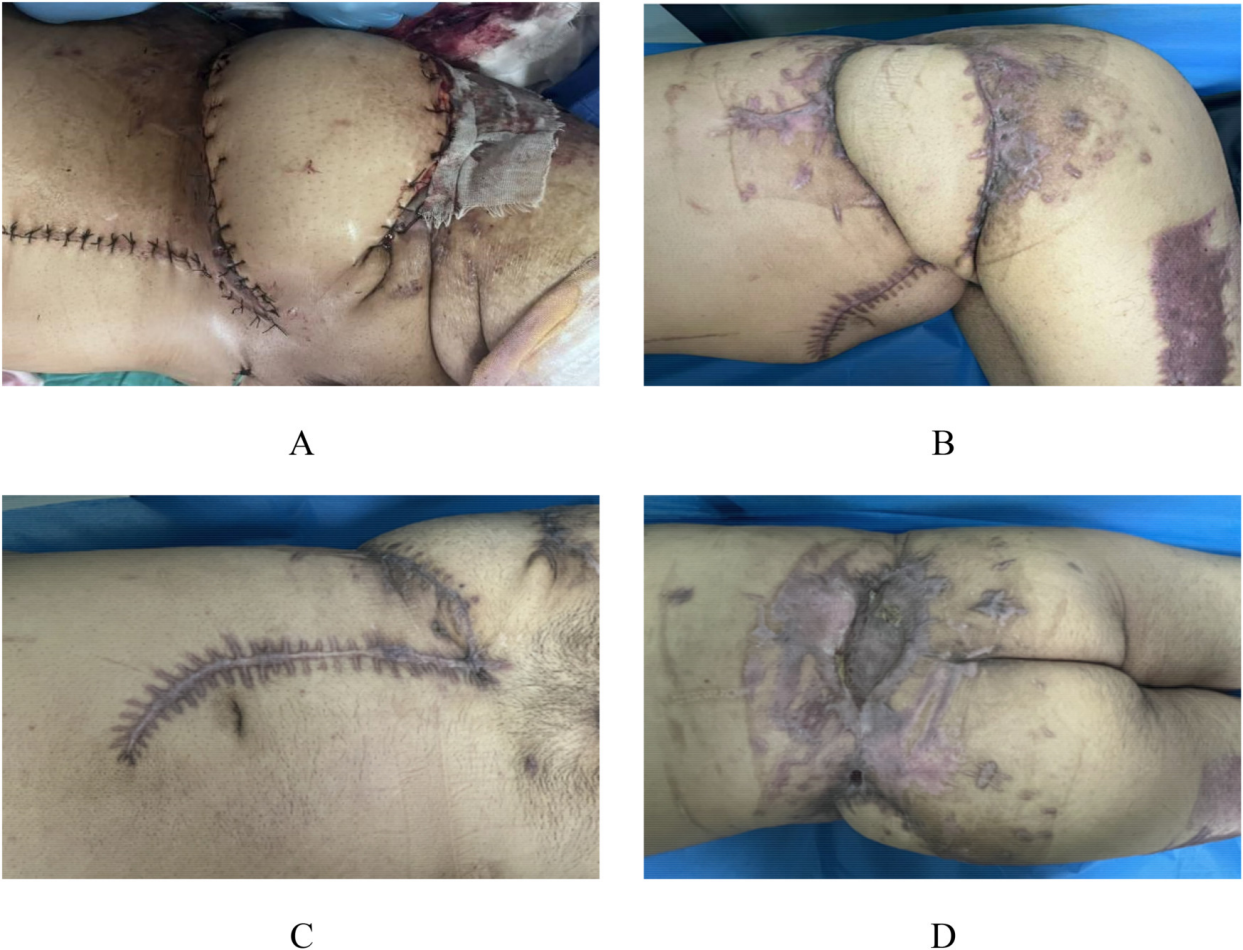
Appearance photos at discharge and 3-month postoperative follow-up. The patient continued to be hospitalized in our hospital for 1 month and then was discharged, with good skin growth after transplantation **(A)** At the re-examination 3 months after surgery, the fracture healed well, and the skin healed well except for a 1 × 1.5 cm fistula in the left posterior lumbar region **(B–D)**.

The patient continued to be remained admitted in our facility for 1 month and then was discharged. The transplanted skin grew well (Figure 5A). The patient was admitted for observation in a local primary hospital and came to our hospital for regular review. At the 3-month postoperative review, the fracture healed well. Except for a 1 × 1.5 cm fistula in the left posterolateral lumbar region, the skin healed well in other areas (Figures 5B–D).

## 3 Discussion

The pathogenesis of MLL is well-established, primarily resulting from high-energy shear forces, blunt trauma, or crush injuries that cause separation of the superficial subcutaneous tissue from the underlying robust deep fascia, thereby creating a potential space at the fascial level. This traumatic disruption concurrently leads to rupture of traversing blood vessels, nerves, and lymphatic channels during the degloving process (8). Research indicates this pathological mechanism correlates strongly with both the presence of large, loosely organized adipose compartments and variations in fascial anchoring patterns (9), explaining the higher susceptibility observed in patients with elevated body mass index. The accumulation of blood and lymphatic fluid within this potential space ultimately combines with liquefied necrotic adipose tissue. For smaller lesions, percutaneous aspiration combined with drainage and compression dressings typically results in spontaneous resolution (10). However, extensive lesions often lead to incomplete fluid evacuation, resulting in pseudobursa formation where hematomas and liquefaction necrosis produce adipose microglobules that may predispose to hematogenous infection. Both primary and secondary fluid collections can compromise local cutaneous perfusion, ultimately causing skin necrosis (11). The current case, involving an obese construction worker sustaining shear-force injury from a swinging heavy object, demonstrates perfect concordance with this established pathophysiological framework.

The reported incidence of MLL in clinical practice remains inconsistent due to their relative rarity and frequent association with high-energy trauma. Muneer et al. (12) documented 183 cases (31.5%) of MLL among 580 pelvic trauma patients, predominantly resulting from motor vehicle accidents and falls from height, with 92.3% occurring in young males. In contrast, Shen et al. (13) reported a lower male predominance (2:1 ratio), suggesting methodological variations while confirming male predilection in high-energy MLL cases. Vanhegan et al.'s (14) systematic review quantified anatomical distribution: greater trochanter (30.4%), thigh (20.1%), pelvis (18.6%), knee (15.7%), lumbar region (6.4%), lumbosacral area (3.4%), abdominal wall (1.4%), calf (1.5%), and head (0.5%). The true clinical incidence remains uncertain as smaller MLL are frequently overlooked (15). Diagnosis primarily relies on physical examination findings including skin mobility on palpation, fluctuance, hypesthesia, and ecchymosis (16), though some cases present solely with localized anesthesia and fluctuance without visible trauma (17). Kottmeier et al. (18) reported 44% missed diagnosis rate (7/16 cases) in their series. While MRI demonstrates highest diagnostic accuracy, its utility is often limited in emergency settings. Nickerson et al. (19) found no significant advantage of radiography, CT, or ultrasonography over clinical examination for acute MLL diagnosis, though these modalities remain essential for therapeutic planning and prognostic evaluation. In our case, the 2-week diagnostic delay was likely due to under-recognition of MLL features at the referring center, where subcutaneous hematoma was misinterpreted, delaying the recognition of MLL.

MLL carry significant infection risks for both soft tissue and bone structures. The patient had a previous diagnosis of type 2 diabetes mellitus, which may potentially affect the patient's infection risk and healing by impairing neutrophil function, reducing tissue perfusion through microvascular disease, and causing metabolic disorders that disrupt collagen synthesis. During the treatment, we strictly controlled the patient's blood glucose to minimize the impact on infection and tissue repair. Shen et al. (13) reported a 19% infection rate (29/153 cases) in their MLL cohort, while Suzuki et al. (20) demonstrated an 8-fold increased relative risk of postoperative infection in femoral head fracture patients with concomitant MLL compared to those without. The pathogenesis of skin necrosis involves two primary mechanisms: direct traumatic disruption of cutaneous and subcutaneous tissues, and ischemia resulting from compromised perfusion to the dermal vascular plexus (21). Early accurate identification of MLL—including precise localization and size assessment—coupled with appropriate intervention strategies is crucial for preventing skin necrosis. The optimal timing for managing MLL with associated fractures remains controversial, requiring careful risk-benefit analysis to minimize postoperative infections (22). Ronceray et al. (23) established open debridement as an effective approach, enabling complete necrotic tissue removal (including fibrotic pseudocapsule excision) while preventing soft tissue infections. Current protocols recommend simultaneous management of open fractures and soft tissue lesions during initial surgery. However, treatment algorithms for MLL with closed fractures remain debated, particularly regarding single-stage vs. staged procedures. Labler et al. (24) advocated wound closure after MLL stabilization followed by secondary fracture fixation to reduce infection risks, while other experts favor concurrent debridement and open reduction/internal fixation (25). For open injuries with neurovascular or tendinous involvement, primary repair is indicated, with vacuum-assisted closure (VAC) proving particularly valuable in cases of extensive skin defects or high tension, especially for graft/flap reconstruction (26). Although emerging technologies show promise (27, 28), their clinical efficacy requires further validation. Our case, which developed osteomyelitis and skin necrosis, was managed through a three-phase protocol: Phase I (muscle debridement, iliac fracture fixation, VAC); Phase II (pelvic reconstruction with PMMA void filling, VAC); and Phase III (complex flap reconstruction with skin grafting, debridement, VAC). This staged approach successfully addressed soft tissue debridement, fracture stabilization, osteomyelitis control, and definitive wound coverage, ultimately achieving bony union and complete cutaneous healing with satisfactory outcomes.

In summary, our study report indicates that MLL is a rare high-energy trauma. During emergency admission, special attention should be paid to whether patients have this concomitant injury, especially obese patients. For patients with MLL complicated by fractures, early radical debridement is necessary. Subsequent staged management of fractures and soft tissues can be carried out. The use of VAC throughout the treatment process may significantly reduce postoperative infection risk.

In clinical practice, early diagnosis of MLL should be alerted in patients with high-energy trauma. For obese patients in particular, clinical manifestations should be combined with imaging examinations to avoid missed diagnosis. For complex MLL complicated with fractures, a three-stage treatment strategy of “debridement-infection control-soft tissue reconstruction” is recommended: thorough debridement and application of VAC for infection control in the early stage, management of osteomyelitis with antibiotic-loaded bone cement in the second stage, and flap transplantation or skin graft repair after wound conditions improve. The application of VAC technology throughout the treatment process can effectively reduce exudation, promote granulation tissue formation, and reduce the risk of postoperative infection. Meanwhile, the impact of underlying diseases such as diabetes on infection control and wound healing should be paid attention to, and multidisciplinary collaboration should be adopted to optimize patient prognosis.

## Data Availability

The original contributions presented in the study are included in the article/Supplementary Material, further inquiries can be directed to the corresponding author.
